# Using routine data to monitor inequalities in an acute trust: a retrospective study

**DOI:** 10.1186/1472-6963-12-104

**Published:** 2012-04-26

**Authors:** Katharine M Langford, Alex Bottle, Paul P Aylin, Helen Ward

**Affiliations:** 1 School of Public Health, Imperial College London, Norfolk Place, London, W2 1PG, UK; 2 Dr. Foster Unit, Department of Primary Care and Public Health, Imperial College London, 1st Floor Jarvis House, 12 Smithfield Street, London, EC1A 9LA, UK

## Abstract

****Background**:**

Reducing inequalities is one of the priorities of the National Health Service. However, there is no standard system for monitoring inequalities in the care provided by acute trusts. We explore the feasibility of monitoring inequalities within an acute trust using routine data.

****Methods**:**

A retrospective study of hospital episode statistics from one acute trust in London over three years (2007 to 2010). Waiting times, length of stay and readmission rates were described for seven common surgical procedures. Inequalities by age, sex, ethnicity and social deprivation were examined using multiple logistic regression, adjusting for the other socio-demographic variables and comorbidities. Sample size calculations were computed to estimate how many years of data would be ideal for this analysis.

****Results**:**

This study found that even in a large acute trust, there was not enough power to detect differences between subgroups. There was little evidence of inequalities for the outcome and process measures examined, statistically significant differences by age, sex, ethnicity or deprivation were only found in 11 out of 80 analyses. Bariatric surgery patients who were black African or Caribbean were more likely than white patients to experience a prolonged wait (longer than 64 days, aOR = 2.47, 95% CI: 1.36-4.49). Following a coronary angioplasty, patients from more deprived areas were more likely to have had a prolonged length of stay (aOR = 1.66, 95% CI: 1.25-2.20).

****Conclusions**:**

This study found difficulties in using routine data to identify inequalities on a trust level. Little evidence of inequalities in waiting time, length of stay or readmission rates by sex, ethnicity or social deprivation were identified although some differences were identified which warrant further investigation. Even with three years of data from a large trust there was little power to detect inequalities by procedure. Data will therefore need to be pooled from multiple trusts to detect inequalities.

## Background

Reducing health inequalities has been an explicit priority in the United Kingdom (UK) for over a decade, informing operational strategy in the National Health Service (NHS) [1], and government policy more widely [2]. The causes of inequalities are varied and include environmental, social and behavioural determinants. The Marmot strategic review of health inequalities *Fair Society, Healthy Lives* described how health inequalities result from wider social inequalities [3]. While the root of inequalities often lies in the broader determinants of health, it is also important to evaluate whether health services play a role in perpetuating or ameliorating existing health inequalities. Even in a universal health care system such as the NHS there is potential for certain groups to receive inadequate care. Health inequalities can refer to differences in health status, outcomes or treatment [4]. Differences in health are often deemed unfair if these health disparities are adversely affecting those who are already socially disadvantaged [5]. This study focuses on exploring to what extent routine data can be used to explore and monitor inequalities in the care provided by an acute trust.

The NHS constitution sets upper limits for waiting times and it is a patient’s right to have treatment within this time [6]. Waiting times can be used as an indicator of access to care. A study looking at total hip replacements found that patients who wait longer have poorer post-operative outcomes [7]. The evidence on inequalities in waiting times from the UK and Europe is in consistent– some studies have shown no relationship between longer waiting times and age, sex or ethnicity [8]. Contrary to this, a European study found that a higher education level was associated with shorter waiting times for elective surgery [9], and Cooper *et al.* found that inequity with regards to waiting times had decreased since 1997 [10]. A study looking specifically at cardiac surgery found that those from more deprived areas were less likely to be classified as urgent, and as such would wait longer for cardiac surgery [11]. Additionally, a systematic review of invasive procedures for coronary heart disease found that inequalities in waiting times and procedures rates existed in the UK [12].

Length of stay is often used as a marker of hospital efficiency and can be difficult to use as an indicator of quality of care [13]. It is the result of many different factors including clinical, socio-demographic and organisational. However, if there are differences in length of stay between socio-demographic groups, the causes of this may need to be investigated. For example, in a study of total knee replacements, those from more socio-economically deprived areas were found to stay longer in hospital despite similar levels of post-operative morbidity and clinical need [14]. It was hypothesised that this may be due to a lack of social support. National studies have found that variation in length of stay could be partially explained by indicators of poverty [15]. This was also shown for specific procedures such as elective colorectal surgery [16], and total joint replacements [14,17].

As post-operative mortality is relatively rare, 28-day readmission rates are often used as an indicator of quality of care, though there is a debate over how useful an indicator it is [18]. A review of studies which looked at readmission rates found that between 9% and 48% of all readmissions could have been prevented and indicated that the patient had received substandard care [18]. An audit of readmissions in an English trust found that a fifth of readmissions were preventable [19]. Looking at readmissions broadly may not be useful as there are many possible confounding variables, however they could be useful if used to identify trusts or areas of care where there are inequalities, where more in-depth local studies could then be conducted [19]. A UK study found that more deprived patients were more likely to be readmitted, though the reason for this was not explained [20]. Research on readmissions has found that, for colorectal surgery, social deprivation was associated with increased readmissions [16].

Inequalities may be due to variation in the quality of care between Trusts serving different populations, or may occur because the quality of care in the same organisation varies according to, for example, the socioeconomic status or ethnicity of individual patients. Monitoring of inequalities on a local level could identify problem areas, such as systematic discharge delays or readmissions for particular groups which could be investigated and tackled by health professionals and managers. Routine datasets, such as Hospital Episode Statistics (HES), provide the potential to monitor inequalities in process and outcome measures of inpatient treatment. For example, Hacker and Stanistreet used HES to explore whether certain groups had longer waiting times for ophthalmology and orthopaedic surgeries [21]. Morgan and Hamm used a waiting list database to examine ethnic inequalities in waiting times for certain procedures [22].

We measured inequalities in access (waiting times), process (length of stay) and outcome (readmission) by age, sex, ethnicity and social deprivation for seven common procedures at a single large acute trust, and examined whether such data could usefully be applied more generally to monitor inequalities at the trust level.

## Methods

### **Hospital Episode Statistics (HES)**

This study used a retrospective design to explore the feasibility of monitoring inequalities within an acute trust. We obtained routinely collected administrative data, HES, from April 2007- March 2010, from the NHS Connecting for Health secondary uses service (SUS). We hold Section 251 National Information Governance Board for Health and Social Care permission to hold these data for research purposes. We also hold South East Local Research Ethics Committee approval to analyse the data. HES have been collected on all patients admitted to NHS hospitals since 1989 and include demographic, diagnostic and procedural data [23]. Three years of data were used to gain the largest numbers of patients whilst minimising additional confounding because of changes over time in coding, medical practice or policy, although coding may have improved over the course of these three years which we cannot control for [24].

HES records represent the finished consultant episode – “a period of admitted patient care under a consultant or allied healthcare professional within an NHS trust” [23]. A stay in a hospital can be made up of one or multiple finished consultant episodes. These were linked together into admissions which is the unit of analysis used here. Transfers in from other hospitals were not included. A proportion of the admissions considered in this study would have ended as transfers, however post-transfer length of stay and readmissions were not considered in this study.

### **Procedures and database inclusions**

Inequalities were examined within procedure groups rather than specialities so that patients undergoing similar procedures were compared to each other. Although patients can have multiple procedures within an admission, patients were grouped by their main operative procedure, the most resource intensive procedure of the admission [23]. This study looked at elective admissions for seven procedures - bariatric surgery, cholecystectomy, coronary angioplasty, primary hip replacement, inguinal hernia repairs, primary knee replacement and mastectomy (for breast cancer in women). These elective surgical procedures were chosen in discussion with service leads to include the most common, and those that were rapidly increasing in volume and those where inequalities have been reported from national data. By including common procedures we would increase the statistical power to identify inequalities should any exist. Non-elective patients, day admissions and patients under 18 were excluded from the analysis to try to reduce the variability in the sample and to take into account some of the case-mix within the procedure groups.

### **Measures of inequalities**

This study explored variations in access, process and outcome measures by age, sex, ethnicity and social deprivation. Data on age, sex, ethnicity and patient postcode are routinely included within HES. Data on ethnicity, however, is often recorded as not stated or not known, though this improved nationally from 24% missing in 2004 to 9% in 2010 [25]. Due to small numbers in some groups, age bands were combined into 3 to 5 groups, based on the distribution of age for each procedure area. Ethnicity categories within HES are based on the ethnic groups used in the 2001 census [23]. Small numbers made it necessary to combine these into four categories – white, Asian, black and other and mixed backgrounds. The Carstairs index of deprivation was used to determine the social deprivation of the postcode of the patient’s home address as a proxy for the patient’s socioeconomic status [23]. The Carstairs index was used in the dataset as it is available at a smaller area level, the lower super output area. The scores in the original dataset were split into five population-weighted quintiles based on the national distribution. These were combined into 2 groups, quintiles 1 to 3 and quintiles 4 to 5, to provide sufficient numbers and enable comparison between the more and less deprived.

In addition to the four socio-demographic variables which were used to explore inequalities, data on comorbidities were used to take case-mix into account. The dataset included a measure of comorbidity. Each patient had a primary diagnosis, any other secondary diagnoses or comorbidities were used to derive a comorbidity score using the Charlson comorbidity index, taking into account both the number and severity of the comorbidities that a patient might have [26]. The weights used were derived from English administrative hospital data [27]. The comorbidity score was dichotomized into a binary variable ‘no comorbidities’ or ‘one or more comorbidities’.

### **Dependent variables: process and outcome measures**

The process and outcome measures of hospital care used were waiting times, length of stay and readmissions. Waiting time is the time between the date on which the patient was put on the waiting list and the date on which they were admitted, and therefore includes any time when an individual might be suspended from the waiting list, a patient does not attend or if a patient is unable to have surgery because of ill health [23]. It is only valid for elective patients with planned admissions; non-elective patients were therefore excluded from all analyses conducted for this study. Length of stay represents the number of days the patient spends in the hospital during their admission. The continuous variables length of stay and waiting times were tested for normality. Common normalising transformations of the data, such as the reciprocal, square root and natural log were unsuccessful and binary variables were therefore created from these continuous variables. Under the NHS constitution patients should not wait more than 18 weeks from referral for treatment, however this could not be used to define a prolonged waiting time as too few people waited longer than this time [23]. 75th percentiles were therefore used for each procedure group. The 75^th^ percentile was also used to define a prolonged length of stay. A similar study looking at waiting times used the median as a cut-off point but we used the 75^th^ percentile as the tail of the distribution was of more interest [21]. 75th percentiles have been used in other studies to define a prolonged length of stay [16]. Readmissions were measured using the derived field of unplanned readmissions within 28 days of discharge. Those patients who died were excluded from the analysis when readmissions were analysed. Readmissions were not explored for inguinal hernia repairs as there were too few readmissions.

### **Data analysis**

The data analysis was conducted using SPSS v.18 (SPSS Inc, Chicago, Illinois, USA). Statistical significance was set at p ≤ 0.01, to take the multiple analyses into account. Descriptive statistics were used to examine the distribution of the variables for the whole population and for each procedure group.

Logistic regression was used for each procedure group to explore the relationship between each of the explanatory variables (ethnicity, social deprivation, age, sex) and the process or outcome measure. Multiple logistic regression was then used to explore the independent effects of the explanatory variables and to adjust for comorbidity.

### **Power and sample size calculations**

Power is the probability of rejecting a false null hypothesis i.e. it is the ability of the test to find an effect that is there. Power calculations were conducted retrospectively for one of the analyses - the relationship between social deprivation and a prolonged wait for coronary angioplasty, as Pell and Pell *et al.*’s 2000 study found a similar relationship [11]. Sample size calculations were also made, using this study as a pilot, to determine how many years of data would be needed to detect an effect.

Power and sample size calculations were calculated retrospectively using G*Power 3.1.2. For logistic regression this program uses the methodology described in Hsieh, Block & Larsen’s 1997 paper [28]. Rather than using complex calculations for logistic regression, this method is based on comparing proportions and then adjusting for a multifactorial model by a variance inflation factor [28].

## Results

### **Characteristics of the population**

The characteristics of the population, including age, sex, social deprivation, ethnicity and comorbidities, are described in Table 1. The waiting time, length of stay and readmission rate varied substantially across the seven procedure areas examined (Table 1). Mastectomy had the shortest median waiting time of 20 days while knee replacement had the longest of 88 days. Those patients undergoing bariatric surgery, cholecystectomy, coronary angioplasty or inguinal hernia repair tended to have a short stay with a median length of stay of 1 day. Length of stay was longer for those undergoing a mastectomy, hip replacement or knee replacement. The procedure with the highest readmission rate was bariatric surgery with 7.7% readmitted within 28 days, and the lowest was inguinal hernia with 3% readmitted.

**Table 1:** Characteristics of Admissions 2007-2010

	**Bariatric Surgery**		**Cholecystectomy**		**Coronary Angioplasty**		**Hip replacement**			**Inguinal hernia**		**Knee replacement**		**Mastectomy**	
**Number of admissions**	543		958		2238		778				1129		1303	
**Number of patients**	533		955		2060		734		872		1018		1104	
**Age Group**	**N**	**%**	**N**	**%**	**N**	**%**	**N**	**%**	**N**	**%**	**N**	**%**	**N**	**%**
18-24	20	3.8	29	3.0	0	0.0	2	0.3	18	2.0	0	0.0	4	0.3
25-34	77	14.2	169	17.6	6	0.3	14	1.8	61	6.8	2	0.2	31	2.4
35-44	180	33.1	177	18.5	50	1.2	48	6.2	105	11.7	11	1.0	142	10.9
45-54	168	30.9	218	22.8	296	13.2	86	11.1	116	12.9	79	7.0	381	29.2
55-64	80	14.7	166	17.3	681	30.4	189	24.3	199	22.1	290	25.7	388	29.8
65-74	18	3.3	134	14.0	801	35.8	239	30.7	225	25.0	427	37.8	237	18.2
75-84	0	0.0	62	6.5	365	16.3	166	21.3	133	14.8	275	24.4	98	7.5
85+	0	0.0	3	0.3	39	1.7	34	4.4	43	4.8	45	4.0	22	1.7
**Sex**														
Male	116	21.4	250	26.1	1680	75.1	346	44.5	819	91.0	349	30.9	0	0.0
Female	427	78.6	708	73.9	558	24.9	432	55.5	81	9.0	780	69.1	1303	100.0
**Deprivation**														
Q1-3 less deprived	170	31.3	281	29.3	1075	48.0	278	35.7	315	35.0	369	32.7	492	37.8
Q4-5 more deprived	370	68.1	675	70.5	1119	50.0	495	63.6	581	64.6	759	67.2	795	61.0
Missing	3	0.6	2	0.2	44	2.0	5	0.6	4	0.4	1	0.1	16	1.2
**Ethnic Group**														
White	287	52.9	574	59.9	1114	49.8	616	79.2	515	57.2	670	59.3	802	61.6
Asian	48	8.8	74	7.7	598	26.7	46	5.9	69	7.7	180	15.9	126	9.7
Black	62	11.4	90	9.4	75	3.4	33	4.2	67	7.4	130	11.5	114	8.7
Mixed & Other	58	10.7	104	10.9	160	7.1	47	6.0	86	9.6	102	9.0	121	9.3
Missing	88	16.2	116	12.1	291	13.0	36	4.6	163	18.1	47	4.2	140	10.7
**Comorbidity**														
No	335	61.7	769	80.3	1177	52.6	588	75.6	720	80.0	811	71.8	1036	79.5
Yes	208	38.3	189	19.7	1061	47.4	190	24.4	180	20.0	318	28.2	267	20.5
**Procedure**														
Gastric bypass	275	50.6												
Sleeve gastrectomy	126	23.2												
Gastric banding	142	26.2												
**Emergency Readmissions**														
No	501	92.3	903	94.3	2118	94.6	721	92.7	873	97	1053	93.3	1235	94.8
Yes	42	7.7	55	5.7	120	5.4	57	7.3	27	3	76	6.7	68	5.2
	**Median**	**IQR**	**Median**	**IQR**	**Median**	**IQR**	**Median**	**IQR**	**Median**	**IQR**	**Median**	**IQR**	**Median**	**IQR**
**Waiting time (days)**	35	15-64	45	21-74	35	18-56	71	43-116	48	21-85	88	53-128	20	10-27
**Length of stay (days)**	2	1-2	1	1-2	1	1-1	5	4-7	1	1-2	5	4-7	4	1-7

The 75^th^ percentiles of waiting times and length of stay that were used to define a prolonged wait or prolonged stay for each procedure are indicated by the upper limit of the inter-quartile range (IQR) in Table 1.

### **Logistic regression**

Univariate and multiple logistic regressions were carried out to determine whether inequalities existed in waiting times, length of stay and readmission rates for the seven procedure groups. Table 2 shows an example of one of these regression models (the others are appended). The adjusted odds ratios for the relationships between age, sex, ethnicity and social deprivation are shown in Tables 3, 4, 5, 6.

**Table 2:** Example of univariate and multiple regression analysis for waiting times for coronary angioplasty

**Independent Variable**	**Level**	**N**	**Univariate analysis**		**Adjusted analysis^$^**	
			**OR (95% CI)**	**p-value**	**OR (95% CI)**	**p-value**
**Age**	18-54	71 (291)	Reference			
	55-64	146 (540)	1.15 (0.83- 1.59)	0.41	1.13 (0.82- 1.57)	0.47
	65-74	159 (666)	0.97 (0.71- 1.34)	0.86	0.97 (0.70- 1.34)	0.83
	75+	89 (336)	1.12 (0.78- 1.60)	0.55	1.10 (0.76- 1.60)	0.60
**Sex**	Male	333 (1355)	Reference			
	Female	132 (478)	1.17 (0.93- 1.48)	0.19	1.15 (0.90- 1.46)	0.26
**Ethnicity**	White	266 (1038)	Reference			
	Asian	146 (574)	0.99 (0.78- 1.25)	0.93	0.87 (0.68- 1.12)	0.27
	Black	14 (71)	0.71 (0.39- 1.30)	0.27	0.58 (0.32- 1.08)	0.08
	Mixed & Other	39 (150)	1.02 (0.69- 1.51)	0.92	0.92 (0.62- 1.37)	0.68
**Deprivation**	Q1-3	197 (867)	Reference			
	Q4-5	268 (966)	1.31 (1.06- 1.62)	0.01	1.32 (1.06- 1.66)	0.02
**Comorbidity**	No	210 (924)	Reference			
	Yes	255 (909)	1.33 (1.07- 1.64)	0.01	1.32 (1.06- 1.64)	0.01

† Significant at p < 0.01 level.

$ Adjusted for age, sex, ethnicity, social deprivation and comorbidity.

**Table 3:** **Adjusted odds ratios for age**^**$**^

	**Bariatric surgery**		**Cholecystectomy**		**Coronary angioplasty**		**Hip replacement**		**Inguinal hernia repair**		**Knee replacement**		**Mastectomy**	
	**Age**	**aOR (95% CI)**	**Age**	**aOR (95% CI)**	**Age**	**aOR (95% CI)**	**Age**	**aOR (95% CI)**	**Age**	**aOR (95% CI)**	**Age**	**aOR (95% CI)**	**Age**	**aOR (95% CI)**
**Waiting time**	**18-44**	1	**18-44**	1	**18-54**	1	**18-64**	1	**18-44**	1	**18-64**	1	**18-54**	1
	**45-54**	0.77 (0.46-1.28)	**45-54**	1.79 (1.16-2.74)†	**55-64**	1.13 (0.82-1.57)	**65-74**	0.74 (0.49-1.12)	**45-54**	0.75 (0.40-1.40)	**65-74**	1.11 (0.80-1.55)	**55-64**	0.65 (0.46-0.92)
	**55+**	0.98 (0.55-1.76)	**55-64**	1.51 (0.95-2.40)	**65-74**	0.97 (0.70-1.34)	**75-84**	0.70 (0.44-1.11)	**55-64**	0.66 (0.38-1.14)	**75-84**	1.02 (0.70-1.48)	**65-74**	0.75 (0.50-1.11)
			**65+**	1.57 (1.00-2.47)	**75+**	1.10 (0.76-1.60)	**85+**	0.71 (0.29-1.75)	**65-74**	0.93 (0.56-1.55)	**85+**	1.07 (0.50-2.29)	**75+**	1.20 (0.74-1.94)
									**75+**	0.78 (0.45-1.34)				
**Length of stay**	**18-44**	1	**18-44**	1	**18-54**	1	**18-64**	1	**18-44**	1	**18-64**	1	**18-54**	1
	**45-54**	1.86 (1.01-3.43)	**45-54**	1.53 (0.82-2.87)	**55-64**	1.33 (0.85-2.09)	**65-74**	1.09 (0.66-1.81)	**45-54**	1.18 (0.53-2.63)	**65-74**	1.45 (0.96-2.18)	**55-64**	0.86 (0.60-1.24)
	**55+**	1.32 (0.60-2.90)	**55-64**	2.30 (1.24-4.28)†	**65-74**	1.62 (1.05-2.49)	**75-84**	3.43 (2.12-5.55)†	**55-64**	1.18 (0.59-2.36)	**75-84**	3.47 (2.29-5.24)†	**65-74**	0.44 (0.26-0.73)†
			**65+**	4.39 (2.51-7.67)†	**75+**	2.08 (1.30-3.34)†	**85+**	9.02 (4.00-20.33)†	**65-74**	1.81 (0.96-3.44)	**85+**	11.62 (5.76-23.47)†	**75+**	2.37 (1.48-3.79)†
									**75+**	2.48 (1.30-4.73)†				
**Readmissions**	**18-44**	1	**18-44**	1	**18-54**	1	**18-64**	1	**18-44**		**18-64**	1	**18-54**	1
	**45-54**	1.47 (0.71-3.04)	**45-54**	1.43 (0.68-3.00)	**55-64**	0.76 (0.41-1.38)	**65-74**	1.80 (0.84-3.84)	**45-54**		**65-74**	1.02 (0.59-1.91)	**55-64**	1.35 (0.74-2.47)
	**55+**	1.00 (0.38-2.67)	**55-64**	1.06 (0.46-2.47)	**65-74**	0.89 (0.50-1.57)	**75-84**	2.86 (1.34-6.07)†	**55-64**		**75-84**	1.34 (0.72-2.49)	**65-74**	0.68 (0.30-1.57)
			**65+**	1.19 (0.54-2.63)	**75+**	0.93 (0.48-1.79)	**85+**	8.08 (2.92-22.38)†	**65-74**		**85+**	1.31 (0.42-4.04)	**75+**	0.85 (0.31-2.36)

† Significant at p < 0.01 level.

$ Adjusting for sex, ethnicity, social deprivation and comorbidity.

**Table 4:** **Adjusted odds ratios and 95% confidence intervals for sex**^**$**^

		**Bariatric surgery**	**Cholecystectomy**	**Coronary angioplasty**	**Hip replacement**	**Inguinal hernia repair**	**Knee replacement**
**Waiting Time**	**Male (Reference)**	1	1	1	1	1	1
	**Female**	0.61 (0.36-1.02)	1.38 (0.94-2.03)	1.15 (0.90-1.46)	1.10 (0.78-1.56)	0.83 (0.45-1.53)	1.13 (0.83-1.53)
**Length of Stay**	**Male (Reference)**	1	1	1	1	1	1
	**Female**	0.81 (0.42-1.59)	0.96 (0.61-1.50)	1.16 (0.87-1.54)	1.26 (0.85-1.87)	1.36 (0.74-2.48)	1.05 (0.75-1.47)
**Readmissions**	**Male (Reference)**	1	1	1	1		1
	**Female**	1.69 (0.63-4.50)	1.62 (0.79-3.31)	1.87 (1.24-2.81)†	0.56 (0.32-1.00)	N/A	0.82 (0.50-1.35)

† Significant at *p* < 0.01 level.

$ Adjusting for age, ethnicity, social deprivation and comorbidity.

**Table 5:** **Adjusted odds ratios and 95% confidence intervals for ethnicity**^**$**^

		**Bariatric surgery**	**Cholecystectomy**	**Coronary angioplasty**	**Hip replacement**	**Inguinal hernia repair**	**Knee replacement**	**Mastectomy**
**Waiting Time**	**White (Reference)**	1	1	1	1	1	1	1
	**Asian**	1.29 (0.63-2.67)	1.73 (1.02-2.93)	0.87 (0.68-1.12)	1.40 (0.73-2.68)	1.09 (0.61-1.93)	1.44 (0.99-2.10)	1.50 (0.98-2.30)
	**Black**	2.47 (1.36-4.49)†	1.26 (0.75-2.11)	0.58 (0.32-1.08)	0.98 (0.44-2.18)	0.88 (0.48-1.61)	1.59 (1.04-2.42)	1.33 (0.84-2.11)
	**Mixed & Other**	0.81 (0.39-1.68)	1.05 (0.63-1.75)	0.92 (0.62-1.37)	0.58 (0.26-1.27)	1.13 (0.67-1.90)	0.84 (0.50-1.43)	0.71 (0.43-1.18)
**Length of Stay**	**White (Reference)**	1	1	1	1	1	1	1
	**Asian**	0.69 (0.22-2.13)	0.62 (0.27-1.41)	1.04 (0.76-1.41)	0.30 (0.09-1.01)	1.02 (0.54-1.93)	0.97 (0.62-1.51)	1.06 (0.65-1.74)
	**Black**	1.38 (0.65-2.96)	1.24 (0.62-2.47)	1.15 (0.61-2.15)	1.52 (0.65-3.52)	1.24 (0.66-2.34)	1.56 (0.99-2.47)	1.22 (0.75-2.00)
	**Mixed & Other**	1.87 (0.84-4.15)	0.80 (0.39-1.64)	1.08 (0.67-1.74)	1.02 (0.46-2.28)	1.09 (0.60-1.99)	1.14 (0.66-1.99)	1.09 (0.67-1.78)
**Readmissions**	**White (Reference)**	1	1	1	1		1	1
	**Asian**	1.26 (0.44-3.60)	1.01 (0.38-2.68)	1.00 (0.63-1.60)	1.64 (0.55-4.92)		0.61 (0.29-1.27)	0.87 (0.36-2.14)
	**Black**	0.92 (0.33-2.58)	0.64 (0.22-1.89)	0.64 (0.19-2.13)	2.22 (0.76-6.52)		1.01 (0.51-2.00)	1.25 (0.55-2.85)
	**Mixed & Other**	1.75 (0.72-4.24)	0.88 (0.36-2.18)	1.80 (0.98-3.33)	1.03 (0.30-3.58)		0.36 (0.11-1.18)	1.09 (0.47-2.55)

† Significant at p < 0.01 level.

$ Adjusting for age, sex, social deprivation and comorbidity.

**Table 6:** **Adjusted odds ratios and 95% confidence intervals for deprivation**^**$**^

		**Bariatric surgery**	**Cholecystectomy**	**Coronary angioplasty**	**Hip replacement**	**Inguinal hernia repair**	**Knee replacement**	**Mastectomy**
**Waiting time**	**Q1-3 (Reference)**	1	1	1	1	1	1	1
	**Q4-5**	0.76 (0.47-1.22)	1.43 (0.98-2.10)	1.32 (1.06-1.66)	1.35 (0.94-1.95)	1.47 (1.01-2.13)	1.02 (0.74-1.39)	0.92 (0.69-1.23)
**Length of Stay**	**Q1-3 (Reference)**	1	1	1	1	1	1	1
	**Q4-5**	1.58 (0.82-3.04)	0.65 (0.43-1.01)	1.66 (1.25-2.20)†	1.55 (1.02-2.34)	1.24 (0.83-1.87)	1.01 (0.71-1.42)	1.37 (0.99-1.89)
**Readmissions**	**Q1-3 (Reference)**	1	1	1	1		1	1
	**Q4-5**	1.36 (0.62-3.01)	1.11 (0.59-2.11)	1.47 (0.96-2.25)	1.42 (0.77-2.63)		1.67 (0.94-2.95)	1.11 (0.63-1.95)

† Significant at *p* < 0.01 level.

$ Adjusting for age, sex, ethnicity and comorbidity.

### **Age**

There was little evidence of inequalities in waiting times in terms of age for any of the procedures. Only for cholecystectomy were those who were 45-54 years old significantly more likely (aOR = 1.79, 95% CI: 1.16-2.74) to have a prolonged wait of over 74 days, compared with those who were 18-44 years old.

There was a largely consistent trend towards a more prolonged stay as age increased with the exception of bariatric surgery. This was most extreme for those undergoing hip and knee replacements, with those over 85 being far more likely to stay over 7 days than younger patients (aOR = 9.02, 95% CI: 4.00-20.33 and aOR = 11.62, 95% CI: 5.76-23.47 respectively).

The only procedure for which there was a significant relationship between readmissions and age was hip replacements. Those who were 75-84 years (aOR = 2.86, 95% CI: 1.34-6.07) and 85+ years (aOR = 8.08, 95% CI: 2.92-22.38) were more likely to be readmitted within 28 days than those who were 18-64 years.

### **Sex**

In general, women did not significantly differ from men with regard to waiting times, length of stay or readmission rates. For coronary angioplasty, however, women were significantly more likely to be readmitted within 28 days compared to men (aOR = 1.87, 95% CI: 1.24-2.81).

### **Ethnicity**

Overall different ethnic groups did not seem to experience significantly different waiting times, lengths of stay or readmissions rates. The only significant difference was that black Caribbean or African bariatric surgery patients seemed to be more likely to experience a prolonged wait of longer than 64 days than white bariatric surgery patients (aOR = 2.47, 95% CI: 1.36-4.49).

### **Social deprivation**

There was little evidence of systematic inequalities by social deprivation. For coronary angioplasty, those from the two most deprived quintiles were 1.66 (95% CI: 1.25-2.20) times more likely to have a prolonged length of stay of over a day.

### **Power calculations and sample size calculations**

Post-hoc power calculations were conducted for the analysis of social deprivation and waiting time for coronary angioplasty. The analysis had a power of 0.47. Based on this study, a sample size of 4,132 would be needed to be able to detect, with 80% power and alpha of 0.01, an odds ratio of 1.3 for the relationship between social deprivation and having a prolonged wait for coronary angioplasty. In this study we used three years of data which gave a sample of 2,238 coronary angioplasty patients. Therefore to get 4,132 patients approximately 6 years of data would be needed.

## Discussion

### **Findings**

This study explored whether routine data can be used to monitor inequalities in an acute trust based on a case study. We found little evidence of inequalities in waiting time, length of stay or readmission rates by sex, ethnicity or social deprivation for common surgical procedures in the trust. We did identify some differences which may warrant further investigation. Overall we conclude that there are challenges in using routine data to monitor inequalities at this level due to limitations in sample size that reduce the power to detect differences. We also identified problems of data validity and relevance in studying inequalities.

We identified most variation in relation to age. Older age groups differed from younger age groups for almost all procedures for length of stay, and for readmissions after hip replacements. This is likely to represent differences in clinical need, as older patients will take longer to recover as well as social support at home contributing to some delayed discharges. We found that people aged 45 to 54 years were more likely to have a prolonged wait for a cholecystectomy; without additional clinical information this is difficult to interpret. It is possible that this group has less of a pressing clinical need compared than those who are 18-44 and therefore waited longer for the procedure.

The only evidence of inequalities by sex was for readmission rates for coronary angioplasty, where women were almost twice as likely to be readmitted. Gender differences have been observed in many different aspects of coronary artery disease and coronary heart disease in terms of epidemiology, diagnosis, treatment and outcomes [29]. Mortality and readmission rates have been found to be higher in women than in men following coronary revascularisation [30,31]. One study, however, found that once baseline clinical risk and body size, a proxy for blood vessel size, were taken into account this was no longer significant [31]. In this analysis we did not look at the diagnosis code for the readmission to see whether it was related to the procedure, this may have provided more clinical information as to the cause of the readmission.

The only significant difference by ethnicity was that black African or Caribbean patients were more likely to have a prolonged wait than white patients for bariatric surgery. This may be due to residual confounding that could not be taken into account. For example we did not have information on primary care trust of origin or clinical factors that could impact on the waiting time.

There was no evidence of systematic inequalities by social deprivation, except for coronary angioplasty. Those from the most deprived areas were 1.7 times more likely to have a prolonged length of stay of over 1 day. There are many explanatory factors that could take account of this. For example, these patients could have a different baseline clinical risk (unaccounted for in our casemix adjustment), although patients from more deprived areas may be less likely to be classified as urgent [11].

### **Strengths and limitations**

The strength of this methodology is that by using easily accessible routine HES data, potential inequalities can be monitored to ensure that the healthcare system is not reinforcing existing inequalities in health. It is a simple way to establish whether certain groups are likely to wait longer, stay longer and be readmitted, adjusting for other factors. The data also allowed for inequalities to be examined by procedure.

The main limitation of using routine data to monitor inequalities on a trust level was that the study was underpowered. Using data from one trust did not provide a large enough sample to detect a medium effect (OR 1.3), though it would have been able to detect larger effects (OR 1.5) for some of the more common procedures. This problem is exacerbated for rarer events such as readmissions. The acute trust that we analysed is one of the largest hospital trusts in England – if it is difficult to use three years of data from such a large trust, this method for monitoring inequalities is likely to be problematic in smaller trusts. More years of data may have given more patients but changes in coding, medicine and population over the years would make the analysis more complex. Pooling data from trusts in a county or neighbouring trusts may make this analysis more feasible – though it is hard to estimate how many trusts you would need to pool together due to the variation in trust size. The small numbers also meant that the grouping of age bands, ethnic and social deprivation groups was necessary, which may oversimplify the patient population and hide inequalities within the groups. Data on social deprivation showed a skewed distribution in our population with a high proportion of people from more deprived areas which will have limited our power to detect a social gradient.

Using routine data also means that the data quality and validity of the data can be problematic. There is, for example, the possibility with an administrative database such as HES that variables have been miscoded [32]. Missing data may be particularly problematic, with 10% of admissions in this study having missing ethnicity codes, and it is possible that this may introduce bias if some groups are more likely to be missing data. However, studies such as this which demonstrate the potential uses of HES data for ethnic monitoring may help to improve the collection of these data [33].

As is the case with many studies using routine data, the data were not collected for this purpose and therefore many variables that would be of interest were not available. For example, though we attempted to take into case-mix by taking into account co-morbidities and by looking at inequalities by procedure, it is very likely that this did not take all the variation of case-mix into account. More data on clinical status may have helped to control for confounding when looking at length of stay and readmissions.

Other limitations included that there is no clear way to dichotomize the outcome variables, length of stay and waiting time. Using 75^th^ percentiles may not inform us of the clinically important differences. For example, the finding that people from more deprived areas were more likely to spend two or more days in the hospital following a coronary angioplasty may not be clinically important. Additionally, multiple tests were performed and therefore it may not be surprising that significant results were found, though a p value of 0.01 was used to try to take this into account.

Even where inequalities were found, using routine data can make it difficult to determine whether these inequalities are inequitable. The main aim of this work was to explore the feasibility of monitoring inequalities not inequities, however the inequalities may be providing equity. More in-depth clinical data, which would not be found in routine administrative dataset may be needed to work out whether these differences are delivering equity or not. For example, this analysis found that older age groups differed from younger age groups for almost all procedures for length of stay. This is most likely to represent a natural difference in clinical need as older patients will take longer to recover; therefore this inequality is not necessarily inequitable. To determine whether it was inequitable, further investigation would be needed into why they are being readmitted and if these readmissions could be prevented.

## Conclusion

It is one of the core aims of the NHS to provide quality care regardless of age, sex, ethnicity and social deprivation. Monitoring of routine data could play an important part in ensure that services are equitable. This study found that there are difficulties in using routine data from one acute trust. Little evidence of inequalities in service provision within the trust were found. It does identify, however, areas where there are statistically significant differences which may warrant further investigation. This methodology shows us that different groups of people may differ in their treatment but it does not tell us whether this difference is fair or what other confounders may be causing this difference. These inequalities should be viewed as a launching point for further investigation by healthcare workers and managers.

## Abbreviations

aOR: Adjusted odds ratio; CI: Confidence interval; HES: Hospital episode statistics; NHS: National health service; OR: Odds ratio; SPSS: Statistical package for social sciences; UK: United Kingdom.

## Competing interests

KML and HW declare no competing interests. AB and PA work for the Dr Foster Unit which receives some funding from Dr Foster Intelligence.

## Authors’ contributions

KML carried out the analysis and drafted the manuscript. AB provided statistical advice and helped to draft the manuscript. PPA participated in the design of the study and helped to draft the manuscript. HW conceived of the study, and participated in its design and coordination and helped to draft the manuscript. All authors read and approved the final manuscript.

## Pre-publication history

The pre-publication history for this paper can be accessed here:

http://www.biomedcentral.com/1472-6963/12/104/prepub
